# Quantifying Antimicrobial Exposure in Dogs From a Longitudinal Study

**DOI:** 10.3389/fvets.2020.00545

**Published:** 2020-11-13

**Authors:** María Méndez, Miguel A. Moreno

**Affiliations:** ^1^Department of Animal Health, Veterinary Faculty, Complutense University, Madrid, Spain; ^2^VISAVET Center, Complutense University, Madrid, Spain

**Keywords:** pets, antibiotics, exposure, metrics, follow-up, Spain

## Abstract

Bacterial resistance to antimicrobials (AMR) is a growing public health threat, and exposure to antimicrobials (AMs) is, on the whole, a major risk factor for the occurrence of AMR. During the past decade, a limited number of studies about AM exposure in dogs have been published, showing a noticeable diversity regarding numerators (AMU), denominators (population at risk), and indicators. The aim of this study is to show that metrics based on the most easily recorded data about treatments and a follow-up design are a promising method for a preliminary assessment of AM exposure in companion animals when more detailed data are not available. To quantify AM exposure, two simple indicators were used: the number of treatments (Ts) per 100 dogs and the number of treatments per 10 dog-years. Overall figures of AM exposure were 194 Ts/100_dogs (480 treatments and 248 dogs) and 18.4 Ts/10_dog-years (480 treatments and 95,171 dog-days), respectively. According to the administration route, AM exposure figures were 126 Ts/100 dogs (305 treatments and 242 dogs) and 12.1 Ts/10_dog-years (305 treatments and 92,059 dog-days) for systemic use and 66 Ts/100 dogs (160 treatments and 242 dogs) and 6.3 Ts/10_dog-years (160 treatments in 92,059 dog-days) for topical use. Since there is no current agreement regarding an indicator for quantifying AM exposure in dogs, in addition to other measures, the simplest indicators based on the most frequently available information should also be reported as a preliminary compromise for permitting a comparative analysis of the different scenarios.

## Introduction

Bacterial resistance to antimicrobials (AMR) is a growing public health threat, and exposure to antimicrobials (AMs) is, on the whole, a major risk factor for the occurrence of AMR; but demonstrating their causal link is challenging (1).

Seminal international conferences in the twentieth century [such as the 1998 Copenhagen recommendations (2)] highlighted the need for an accurate measurement of antimicrobial use (AMU) in humans and animals due to the role of AMU as a key driver of AMR. Shortly afterwards, several influential papers were published in the veterinary field (3–5) stressing both approaches and weaknesses when dealing with this topic. More recently, Collineau et al. (6) summarized this subject focusing on the expected use of AMU metrics.

In the veterinary field, AMU is the most common approach for measuring exposure to AMs, and consequently, numerous AMU metrics have been proposed (7). Most of them are based on the amount of AMs (sold, prescribed, or administered), delivered as raw data (weight of AMs) or standardized for correcting differences in posology (dosage and treatment duration) among both AMs and animal species [for instance, defined daily dose for animals [DDDA] and defined course dose for animals [DCDA], among others].

Curiously, the number of DDDAs indicates the number of days of AM usage, and the number of DCDAs expresses the number of individuals treated; nevertheless, metrics directly computing the days of treatment or treated animals are seldom reported.

These diverse AMU metrics are employed as the numerator for elaborating indicators using a measurement of the population at risk as the denominator. A summary of numerators, denominators, and indicators has been produced (7).

Regarding the design of the studies aimed to assess AMU, the ecological approach is used for international organizations delivering supranational or country-level data [like those reported by the European Medicines Agency in the ESVAC reports (8)]. In this case, different data sources for numerators and denominators are used.

The research studies designed for gathering data for specific animal species are usually retrospective longitudinal studies since data comprise AMU for a study period belonging to an animal population. For food-producing animals, where a closed population model can be used, farms are the natural study units providing records of both AM treatments and a number of animals at risk (including movements). Nevertheless, in the case of companion animals, we do not have groups of animals (except breeding kennels, boarding kennels, and animal shelters), and veterinary practices are the best data source, although the population at risk is not as accurately defined.

During the past decade, a limited number of studies about AM exposure in dogs have been published, showing a noticeable diversity regarding numerators (AMU), denominators (population at risk), and indicators (Table 1).

**Table 1:** Published data of antimicrobial (AM) exposure in dogs.

**References and country**	**Data source**	**Design/period**	**Numerator**	**Denominator (available study size)**	**Indicator**
Radford et al. (9) UK	SAVSNET, clinical data, Sick animals	Three months	Consults with AM	Consults (15,727)	% of consults involving systemic AMs
Mateus et al. (10), UK	Veterinary practices data	January 1, December 31, 2007	A/P^a^ of AMs	Dogs (34,928)	% of dogs with A/P of AMs
Escher et al. (11), Italy	Clinical paper forms	Cross-sectional-−2000–2007	AM prescriptions	Clinical forms (688)	Prevalence of prescriptions
Buckland et al. (12), UK	VetCompass, electronic patient records (EPR)	Two years	AM event	Dogs (963,463)	• % of AM events • Overall quantity of AMs used
Singleton et al. (13), UK	SAVSNET, electronic health records (HER)	1 April 2014 /31 March 2016 (2 years)	• Consultations with AM • Dogs with an AM prescription	• HER (918,333) • Dogs (413,870)	• % of consultations where at least one AM agent was prescribed. • % of dogs prescribed with an AM agent
Hardefeldt et al. (14), Australia	Pet insurance files	2013 to 2017 (4 years)	A/P of a systemic AM and of a systemic AM with a high-importance rating	• Dogs (222,069) • Dog-years (813,172)	• Average proportion of animals exposed to AMs (n° per 1000 dogs) • Incidence rate of exposure to AMs (prescription by 10 dog-year)
Hopman et al. (15), the Netherlands	Veterinary practices data	2012, 2013, 2014	AVMP^b^ procurement data used for calculation of DDDAs^c^	228,000 dogs (110 clinics)	• No. of DDDA clinic/year. Theoretical number of days per year an animal (dog, cat, or rabbit) was treated with AVMPs in the clinic concerned
Joosten et al. (16), Belgium, Italy, and the Netherlands	Veterinary practitioners	Cross-sectional—January 2015–February 2016	Treatment duration X long-acting factor × 100 animals at risk	No. of days at risk (151 dogs)	Treatment incidence (TI) (No. of DDDca^d^/100 days at risk/animal
Hurd et al. (17), Australia	Electronic patient records	2013–2017	Consultations with AM	Total consultations (3,263,615)	AMs dispensed per 1000 consultations

a *Administration/Prescription*.

b *Antimicrobial Veterinary Medicinal Products*.

c *Defined Daily Doses Animal*.

d *Defined Daily Dose for companion animals*.

The aim of this study is to show that metrics based on the most easily recorded data are a promising method for a preliminary assessment of AM exposure in companion animals.

## Materials and Methods

### Sampling Design

Two web-based calls for veterinary practitioners attending dogs in the Autonomous Community of Madrid were launched with the collaboration of the Association of Companion Animals Veterinarians of Madrid (AMVAC) during November and December 2017.

In addition, veterinary practices previously collaborating in a prior study (18) were invited to participate in the study.

In all cases, veterinarians were left blind regarding the main subject of the study (AMU quantification) and were asked by letter if they could provide the full 2016 case history of 10 dogs regularly attending their veterinary practices, as well as the last record of 2015 (a non-illness visit) and the first record of 2017.

### Data Collection and Storage

Data were collected and sent by the veterinarians (usually as a file submitted electronically) or collected at the veterinary practice by the first author.

Data were stored in two spreadsheets (Microsoft Excel). The first contained animal data (date of birth, name, sex, breed) and a summary of the visits. Each visit to a veterinary practice was classified as a non-illness visit (healthy animal going for vaccinations, health checks, or other procedures) or an illness visit (sick animals for any condition including postsurgical visits). The total number of visits, both non-illness and illness, were compiled. In addition, illness visits where an AM was used or prescribed were also accumulated. This spreadsheet also contained data regarding the date and health status of the last visit in 2015 and the date and health status of the first visit in 2017.

The second spreadsheet contained data of AMs, including dog, date, commercial name, active substance, administration route, dosage, duration, and treated condition. Administration routes were grouped as systemic (oral and parenteral routes) and topical (administrations on skin, ears, eyes, etc.).

No personal data from owners or veterinary practitioners (except postal code for veterinary practices) were recorded.

### Quantification of Antimicrobial Exposure

To quantify AM exposure in dogs, two metrics were used: the number of treatments (Ts) per 100 dogs (d) and the number of treatments per 10 dog-years (d-y).

Treatments were used for both numerators and count both AM administration at the veterinary practice and AM prescriptions for at-home use. Treatments starting at the veterinary practice followed by home administration were considered two events.

We used the same numerator for both indicators, including all the AM treatments recorded, irrespective of the number of treatments per dog and of their duration.

Denominators were dog at risk to be AM-treated and dog-year at risk to be AM-treated (sum of the follow-up periods computed as days and then transformed to years).

For the denominator of the second indicator, and assuming that dogs under an AM treatment remain at risk for another AM treatment, the risk period for each dog was the entire follow-up period.

## Results

A retrospective follow-up study was conducted with a convenience sample of 28 veterinary practices. The timeframe of the data set ranged from February 2015 to November 2017.

### Study Population

From the 28 veterinary practices participating in the study, case histories from 279 dogs were obtained. Thirty-one of them were removed; 3 were duplicates from the same dog, 12 were case histories where a non-illness visit for entering the study was not provided, 15 were case histories without visits in 2016, and 1 was a case history with a follow-up period shorter than 3 months.

Finally, 248 dogs were included in the study. One hundred thirty (52.4%) of them were male, and 118 (47.6%) were female.

Breed was recorded for 231 dogs. About 48 different breeds were documented, including crossbreeds. The most recorded breed was crossbred (48 dogs), followed by Yorkshire terrier (20 dogs).

Date of birth was included in 223 case histories and was used for establishing the age (age was calculated by subtracting the reported date of birth from the date of the first appointment). Mean and median age were 5.4 ±3.6 and 4.8 years of age, respectively, ranging from 0.3 to 15.9 years of age (interquartile range 2.3–8.5).

A total of 95,171 days of follow-up were computed from these 248 dogs. The mean and median follow-up times were 384 ±90 and 382 days, respectively, ranging from 121 to 731 days (interquartile range 345–427).

### Visits

A total of 2,148 visits from case histories of 248 dogs were included in the study. The mean number of visits per dog was 8.7 ±6.5 (range, 1 to 49 visits per dog); 1,079 (50.3%) of these were non-illness visits, and 1,069 (49.7%) were illness visits.

Seventeen of the 248 dogs have no records of non-illness visits. The mean number of non-illness visits per dog was 4.4 ±3.4 (range, 0–23). Regarding illness visits, 45 dogs had no records. The mean number of illness visits per dog was 4.3 ±5.0 (range, 0–39 visits).

In addition, the number of illness visits when an AM was prescribed or used was recorded as 423 visits from 157 dogs. The mean number of illness visits with AM per dog was 1.7 ±2.2 (range, 0–14).

### Antimicrobial Exposure

As explained before, to quantify AM exposure, two simple and easily calculable indicators were used: the number of treatments (Ts) per 100 dogs (d) and the number of treatments per 10 dog-years (d-y).

Overall figures of AM exposure were 194 Ts/100_dogs (95% C.I., 177/100–212/100_dogs) (480 treatments and 248 dogs) and 18.4 Ts/10_d-y (95% C.I., 16.7/10–20/10_d-y) (480 treatments and 95,171 dog-days), respectively.

The administration route was explicit on 385 records, and for an additional 80, it was extracted based on the commercial name of the medical product. Thus, this information was available for 465 treatments corresponding to 151 dogs. According to the administration route, AM exposure figures were 126 Ts/100_dogs (95% C.I., 112/100–141/100_dogs) (305 treatments and 242 dogs) and 12.1 Ts/10_d-y (95% C.I., 10.8/10–13.5/10_d-y (305 treatments and 92,059 dog-days) for systemic and 66 Ts/100_dogs (95% C.I., 56/100–77/100_dogs) (160 treatments and 242 dogs) and 6.3 Ts/10_d-y (95% C.I., 5.4/10–7.4/10_d-y) (160 treatments and 92,059 dog-days) for topical use.

AM exposure figures per administration route and most frequently used active substances are presented in Table 2. For producing these figures, data were provided from 464 treatments corresponding to 150 dogs. Figures are lower when both administration route and active substance are included because in two records for one dog, only data about administration route were available.

**Table 2:** Antimicrobial exposure from a 1-year follow-up study of 248 dogs (95,171 dog-day) in Madrid, Spain.

**Antimicrobials and administration route**	**ATCvet codes**	**No. of treatments**	**No. of treated dogs**	**Treatments per 100 dogs**	**Treatments per 10 dog-year**
All		480	157	194	18.4
Systemic^*^:		305	115	126	12.1
· Amoxicillin clavulanate^**^	QJ01CR02	69	42	29	2.8
· Amoxicillin / ampicillin^**^	QJ01CA04 QJ01CA01	68	40	28	2.7
· Cefalexin^**^	QJ01DB01	17	11	7	0.7
· Cefovecin^**^	QJ01DD91	13	11	5	0.5
· Metronidazole^**^	QJ01FA99 QJ01XD01	52	34	22	2.1
· Enrofloxacin^**^	QJ01MA90	14	9	6	0.6
· Marbofloxacin^**^	QJ01MA93	9	8	4	0.4
· Sulphonamides^**^	QJ01EW13 QJ01EQ30	15	8	6	0.6
Topical^*^		160	82	66	6.3
· Neomycin^**^	QS01AA30 QS01AA03 QS02AA07 QS02AA57 QD06AX04	40	28	17	1.6
· Tobramycin^**^	QS01AA12	25	19	10	1
· Polymyxin B^**^	QS02AA11 QS02AA57	22	19	9	0.9
· Marbofloxacin^**^	QS02AA	22	11	9	0.9
· Florfenicol^**^	QS02AA	15	8	6	0.6

* *Denominators for indicators were 242 dogs and 92,059 dog-day, respectively*.

** *Denominators for indicators were 241 dogs and 91,249 dog-day, respectively*.

Among AMs for systemic use, beta-lactams, metronidazole, and fluoroquinolones were the families most recorded, whereas by the topical route, they were aminoglycosides, fluoroquinolones, polymyxins, and phenicols.

## Discussion

### Scope

The analysis of the most frequently used AMs in dogs, including administration routes or other related features, has been addressed in several papers (10–13, 18–20), showing that amoxicillin-clavulanate was, by far, the most frequently used by the systemic routes (10–13, 18, 19). For performing these studies, a population of clinical histories recording AM treatments or AM-treated animals is sufficient as the denominator. Although this information is valuable, this approach does not account for a population at risk, precluding the assessment of AM exposure.

### Data

Although the data used for constructing the above explained indicators for AM exposure have been showed, some decisions must be detailed.

For computing the numerator, we chose the number of treatments since this information was always recorded. Special cases were sequential treatments (starting at the veterinary practice and followed at home) and simultaneous treatments (use of AM combinations). As explained before, we computed two events for sequential treatments since many times the AM prescribed for administration at home was different from the one used at the veterinary practice. Simultaneous treatments were computed as a single treatment for calculating the overall exposure indicators, although active substances, except amoxicillin plus clavulanic acid, were segregated for presenting the exposure data per active substance (Table 2). Long-acting products were computed as one event since the duration of the treatment was not considered for constructing the numerator.

In the case of the denominator counting animal time at risk of AM treatment, it was built by adding the duration of the whole observation time of each enrolled dog. Because treatment duration was not always recorded, it was not subtracted from the denominator. Besides, longer observation times usually belong to healthy dogs attending veterinary services once a year for health checks. Consequently, this denominator probably was overestimated.

Taking together the convenience sampling, the low sampling size, and the above-mentioned circumstances, the extrapolation of our figures to the entire country is not easy to assess. Nevertheless, our feeling is that the veterinary prescription of AMs for dogs is very similar among the Spanish practitioners, irrespective of the geographical area, but we do not know published studies to support this statement.

### Data Sources and Metrics for AM Exposure Assessment

In food animals, where animals are raised by groups and information regarding medication is systematically recorded (mandatory for farmers in many countries), the quantification of both AM exposure and population at risk is easier than in the case of companion animals, where these data are scarce or are not recorded at all.

Since pet owners have no obligation to record this information, records of veterinary practices (case histories) are the only putative source for AM data; but many differences exist between companion animal veterinarians regarding mandatory recording of data for prescriptions or data storage procedures (paper or electronic databases). For instance, in Spain, all the prescriptions of AMs must be issued electronically as from January 2019, but only in the case of farm animals. As stated by Joosten et al. (16), “Currently there is no binding European policy that requires countries to report their veterinary AMU for companion animals. Yet, this will become mandatory for all member states of the European Union by 2030 at the latest (21).”

In some countries, like the Netherlands, “AMs for veterinary use are sold to companion animal owners (or farmers) by veterinarians exclusively” (15) and, consequently, “antimicrobial procurement data are supposed to reflect the total amount of AMs used in animals,” becoming an additional data source for AMU metrics based on the amount of AMs. The number of units of AM products purchased by owners was also used in the UK (3) for quantifying the kilograms of AMs. Nevertheless, this approach is not applicable in Spain.

Another putative source of data of AMU in dogs is the full dosage in clinical records. Among the studies summarized in Table 1, Buckland et al. (12) used the dosage data existing in the VetCompass database for the calculation of the quantity of AMs used. Joosten et al. (16) used data provided by practitioners (the commercial antimicrobial name, the frequency of administration [per day], and the duration of treatment) and data from the Summary of Product Characteristics (SPC) as sources for AMU indicators. Nevertheless, in our experience, the complete data set of a prescription (including dosage and duration) is not always recorded in our country, precluding any approach for calculating the amounts of AMs based on these data. For instance, from the 480 AM treatments recorded in this study, only 56 (11.7%) have the full posology data (dosage and duration). Compliance with SPC data will help to solve this gap of data, but we are not confident that Spanish vets habitually use the SPC data for prescription.

However, almost all case histories of companion animals usually had some information, mostly including the active substance or the medicinal product, that allows us to identify an AM treatment, and these data can be used for AM exposure calculations based on treated animals. Although this procedure does not take into account whether the dosage is correct or not, it has the advantage that real information about the number of treated animals is used. Consequently, metrics using AM treatments or treated animals as the numerator are a good alternative for a preliminary quantification of AM exposure when more detailed information is not available.

The first option (proportion of appointments where an AM has been administered or prescribed) has been applied in some studies attaining figures of 35.5% (9) and 18.8% (13) in UK and 14.5% (17) in Australia, whereas our figure, 19.7% (423/2148), was in between.

The second one (proportion of dogs with a prescription or administration of AMs) has been also reported, but with different study periods. This indicator should be higher when using a follow-up design compared to the cross-sectional one, since the longer the follow-up period, the higher the probability that a dog was AM treated. It is also interesting to note that this indicator has the same interpretation as those based on DCDAs. Figures of 45.1% (10) (1-year period), 25% (16) (1-year period), 28.4% (13) (2-year period), and 18.2% (14) (4-year period) have been provided, whereas our figure was higher (63.3% [157/248 in a 1-year period]). Nevertheless, it is not clear that the mentioned periods can indicate a follow-up of dogs (see the paragraph below).

### Study Design

A cross-sectional design was indicated in some studies (11, 16) and probably also used in others (9, 12, 13) since follow-up of dogs is not mentioned. Nevertheless, this approach requires a high sample size if the outcome of interest has low prevalence. Bearing in mind that animals treated with an AM remain at risk to be treated with other AMs if they suffer a new bacterial disease, a longitudinal design computing multiple occurrences of AM treatments is a good approach for the assessment of AM exposure. In addition, if the duration of the follow-up period by dog is recorded, a denominator based on dog-days units (as in the classical incidence rate used by epidemiology) can be computed providing an alternative indicator. Indeed, for open populations and different follow-up periods, the preferred option for the denominator is computing animal-time units. This approach calculating the incidence rate of exposure to AMs was used in Australia by Hardefeldt et al. (14), obtaining a value of 5.8 prescriptions per 10 d-y, whereas our data were also higher (18.4 treatments per 10 d-y).

### Metrics Based on DDDAs

For the numerator, the calculation of metrics based on DDDAs requires information regarding amounts of AMs used (obtained from packages or from prescriptions), standard dosage from the SPC, and animal weight (from animal records or standard weight tables). This approach has been used in some studies reporting figures corresponding to the number of DDDAs per year from 2.22 to 1.88 in the Netherlands (18) and 3.3 in Belgium, Italy, and the Netherlands (16). Bearing in mind that DDDAs are the number of days per year a dog was treated with AMs (15), an alternative calculation procedure can be applied from our data. In our study, treatment duration was available for 192 (40%) treatments; nevertheless, data from the SPC could be assigned for up to 415 (86.5%) treatments; 279 of these were systemic treatments totalizing 1,834 days and 136 topical treatments (1,140 days). For long-acting products, treatment duration was established according to the SPC and considering the time for a second administration (14 days for cefovecin-containing products and 2 days for amoxicillin-containing products). Extrapolating these data to all the systemic and topical treatments, respectively, the supposed numbers of DDDAs per year in our study were 8.1 and 5.3, for systemic and topical use, respectively. It is interesting to note that the primary indicator used by Joosten et al. (16) is treatment incidence (TI), “which resembles the percentage of a full year that the animal has been treated with a standard dose of AMs,” that is, a metric based on the yearly proportion of days with treatment.

### Antimicrobials Used and Administration Route

Although the main objective of our study using population at risk as the denominator was not to specifically assess the most frequently used AMs, our data confirm, like others, that the above-mentioned ranking in dogs is led by amoxicillin clavulanate (14, 16, 17). Nevertheless, the use of different reporting criteria for the administration route (all AMs aggregated or segregated into systemic vs. topical) or AMs (by active substance or grouped by critical importance or choice) also makes it more difficult to compare published data.

Finally, there is no current agreement regarding an indicator for quantifying AM exposure in dogs, precluding the assessment of data from different sources. In addition to other well-established measures in other animal species, the simplest indicators based on the most frequently available information should be also reported as a compromise for permitting a preliminary comparative analysis of the different scenarios.

## Data Availability Statement

The raw data supporting the conclusions of this article will be made available by the authors, without undue reservation.

## Author Contributions

MM performed sampling and a data analysis and revised the drafted manuscript. MAM designed the study, revised the data analysis, and drafted the manuscript. All authors contributed to the article and approved the submitted version.

## Conflict of Interest

The authors declare that the research was conducted in the absence of any commercial or financial relationships that could be construed as a potential conflict of interest.
